# The Earliest Sign of Consumption

**Published:** 1874-02

**Authors:** 


### THE EARLIEST SIGN OF CONSUMPTION.

A QUICK pulse and a short breath, continuing for weeks together, is the great alarm bell of forming consumption; if these symptoms are attended with a gradual falling off in flesh, in the course of months, there is no rational ground for doubt, although the hack of a cough may never have been heard. Under such circumstances, there ought not to be an hour's delay, in taking competent medical advice.

The vast mass of consumptives die, not far from the ages of twenty-five; and this, in connection with another fact, that consumption is several years in running its course, suggests one of the most important practical conclusions yet announced, to wit:

In the large majority of cases, the seeds of consumption are sown between the ages of sixteen and twenty one years, when the steadily excited pulse and the easily accelerated breathing, may be readily detected by an intelligent and observant parent, and should be regarded as the knell of death, if not arrested, and yet it is easily, and uniformly done, for the Spirometer will demonstrate the early danger, and the educated physician will be at no loss to mark out the remedy.

The quick pulse and short breath go together; rather "*easily put out of breath*," is the more common and appropriate expression. Ordinarily, persons breathe once, while the pulse beats four times; this is an approximative average, a general result. A person in health breathes seventeen times in a minute, and during that time, the pulse numbers sixty eight strokes. A person decidedly consumptive, breathes from twenty to twenty-four times in a minute, the pulse being proportionably rapid. A man whose pulse is among the nineties, with a breathing which corresponds, lasting for weeks, may with great uniformity be pronounced to have unmistaken consumption. And even here, the permanent arrest of the disease is quite a probable thing, if men could only be induced to act wisely, promptly, and energetically. But unfortunately such is not the case; nine out of ten are led away with the hope that it may be something else, that it is only Bronchitis, and this is confirmed in their own judgment by two facts, they have no pain in the breast, and they triumphantly strike upon it with their whole force, as a demonstration of the soundness of the lungs; and this other feeling, equally fallacious comes to their aid, the prominent trouble is a mere tickling at the bottom of the neck, at the little hollow there. They should remember that no Bronchia are there, it is the windpipe. Bronchitis is situated in the branches of the windpipe, and it begins to divide into branches below that spot. That little hollow place is the telegraphic station, as well for the distant lungs as the Bronchia. The news comes from afar; that is the point of enunciation only. It is the news of mischief in the lungs, that something is there which requires removal, which is working harm and may breed death; and it does breed death. That very tickling at the little hollow, exciting cough for months together, is the forerunner of consumption in perhaps, at a moderate calculation, four times out of five. If a person could be amused at such a serious symptom, the physician would be, at the very indifferent, unconcerned air and tone and gesture with which the patient often announces this symptom, "Doctor, I have Bronchitis, I believe, a trifling little tickling at the bottom of the throat here; I wish you would give me something to take it away. I'm not sick at all, I feel as well as I ever did in my life, all except this kind of itching here." Upon a closs cross questioning, a large amount of undiscovered truth will be elicited in almost every instance, of symptoms dated many months and even years before. If then, a patient for himself, or for his child, has any apprehension of the disease, let the family physician be requested to notice the pulse with care and accuracy, at different hours of the day, not within half an hour of active exercise, or within two hours after a regular meal, and if the invariable report be preternatural excitement, there is ground for alarm, in proportion to the intensity of that excitement.

